# Machine-Learning-Based Carbon Dioxide Concentration Prediction for Hybrid Vehicles

**DOI:** 10.3390/s23031350

**Published:** 2023-01-25

**Authors:** David Tena-Gago, Gelayol Golcarenarenji, Ignacio Martinez-Alpiste, Qi Wang, Jose M. Alcaraz-Calero

**Affiliations:** School of Computing, Engineering & Physical Sciences, University of the West of Scotland (UWS), High Street, Paisley PA1 2BE, UK

**Keywords:** hybrid vehicles, IoT, CO_2_, LSTM

## Abstract

The current understanding of CO_2_ emission concentrations in hybrid vehicles (HVs) is limited, due to the complexity of the constant changes in their power-train sources. This study aims to address this problem by examining the accuracy, speed and size of traditional and advanced machine learning (ML) models for predicting CO_2_ emissions in HVs. A new long short-term memory (LSTM)-based model called UWS-LSTM has been developed to overcome the deficiencies of existing models. The dataset collected includes more than 20 parameters, and an extensive input feature optimization has been conducted to determine the most effective parameters. The results indicate that the UWS-LSTM model outperforms traditional ML and artificial neural network (ANN)-based models by achieving 97.5% accuracy. Furthermore, to demonstrate the efficiency of the proposed model, the CO_2_-concentration predictor has been implemented in a low-powered IoT device embedded in a commercial HV, resulting in rapid predictions with an average latency of 21.64 ms per prediction. The proposed algorithm is fast, accurate and computationally efficient, and it is anticipated that it will make a significant contribution to the field of smart vehicle applications.

## 1. Introduction

Vehicles are a major source of pollution, accounting for a total of 25% of annual CO_2_ emissions worldwide [1]. In the last decade, the adoption of hybrid vehicles (HV) on a global scale has proven the reduced levels of CO_2_ emissions generated by HVs in comparison with conventional vehicles. This has sparked interest in industry and academia in finding ways to optimise and reduce vehicle emissions. When it comes to measuring CO_2_ concentrations, a portable emissions monitoring system (PEMS) is the preferred choice, since they are an accurate and reasonably portable. Nevertheless, the usage of this equipment incurs high costs, and therefore, it is mainly used by car manufacturers and environmental regulation entities. It us almost impossible to use for researchers and investigators.

Recent developments in machine learning (ML) mechanisms have led to the creation of pollution predictors. However, these have primarily been focused on conventional internal combustion engine (ICE) vehicles. The complexity and constantly changing nature of power-train sources, coupled with several factors involved in determining the CO_2_ emissions from HVs, make the prediction a challenging task for traditional ML algorithms. A comparative analysis has been summarised in Table 1 to contrast the published results related to CO_2_ emission prediction in automotive applications.

In [2], a real-time cloud-based in-vehicle air quality monitoring system was developed to predict the current and future cabin air quality. Three predictive ML algorithms—linear regression (LR), support vector regression (SVR), and multilayer perceptron (MLP)—were applied for prediction. SVR had the best performance in terms of R^2^ score in their studies when compared with the LR and MLP models. However, our study is focused on the prediction of CO_2_ exhaust concentrations emitted by HVs.

In [3], CO_2_ concentrations were predicted using a tuned gradient boosting regression (GBR) model, which achieved a higher (92%) accuracy compared to other ML algorithms, such as MLP and xtreme gradient boosting regression (XGBR). Focusing on fuel consumption will be their future work for developing a more robust model. In [4], the GBR model was used to predict the emissions of nitrogen oxide (NOx) and carbon dioxide (CO_2_) and the fuel consumption in urban, suburban and highway areas in a conventional diesel-fuelled vehicle. Data collection was performed using a PEMS. Two routes were used to perform the experiments. The model was trained in the first route data and was used to predict the emissions of the second route. The best CO_2_ model had R^2^ scores of 0.98, 0.99 and 0.99 for the various driving patterns. Predictions for the second route were R^2^ scores of 0.79, 0.82 and 0.83, respectively.

In [5], the exhaust emissions and fuel consumption of a HV were tested using a GBR model. However, in contrast to our study, the predictive model only considered the data corresponding to when the ICE was turned on, and therefore, the authors did not contemplate the power train state transitions in HVs. In [12], air pollutant concentrations were modelled using an ANN model for three different traffic volume predictors. Moderate results could be achieved for all CO_2_ concentration levels. All three input variable options (sound, traffic and time) are suitable for modelling various air pollutant concentrations.

In [6], a comparative analysis of LSTM, DNN and CNN was conducted regarding predicting CO_2_ emissions from two light-duty diesel-fuelled vehicles. The dataset was captured from the OBD2 port. The LSTM model outperformed the other models, achieving an RMSE score of 9.30. Plus, the authors analysed the effect of noisy data in the input data, showing that its presence can negatively affect a model’s accuracy by up to 30%.

In [7], several ML techniques used to estimate the CO_2_ emission rate in hybrid vehicles in comparison to traditional micro and macro estimation models. The Gaussian process regression (GPR) model outperformed the rest, achieving a maximum level of accuracy of 69%. As the authors conclude, due to the variability of CO_2_ emission levels from a hybrid vehicle, there is a problem with the existing emission models, as they do not provide reliable results. Our research here provides a contribution to address this phenomenon.

In [9], the authors propose a parallel attention-based LSTM (PA-LSTM) for building an emission prediction model for several vehicle pollutants using PEMS. In this model, the two-layer attention spatial encoding mechanism accelerated the convergence speed of the model to reduce the training time. The results show that the PA-LSTM performed better than any other ML mode, reaching 94.6% accuracy.

In [10], the CO_2_ emissions of a diesel-fuelled vehicle were estimated by means of different ML models. The authors examined the a novel relationship between the movement of construction equipment chassis and the emission rates generated from them. Results show that the best algorithm was RF, obtaining an accuracy level of 94%.

In [11], four different ANNs were studied for predicting the mass-based pollution rates of diesel-fuelled passenger vehicles. Among the analysed models, the ANN that showed the best results was the one trained with the engine speed, engine torque, vehicle speed, coolant temperature and air–fuel ratio.

To sum up, almost all the current literature focuses on prediction of CO_2_ emissions using ICE passenger vehicles. Only two works used a HV during the experiments, of which only the work done in [7] studied the prediction during the vehicle’s power-train switch-overs, which is the biggest challenge in HVs. The results reflected in this study are about 27% less accurate than the average score obtained by the rest of the studies present in the literature. Hence, it is clear that the complex behaviour of HV lowers the accuracy of CO_2_ emission prediction to a large extent when using current ML-based mechanisms.

To tackle this problem, we designed, implemented and evaluated a long short-term memory-based (LSTM) model named UWS-LSTM for predicting CO_2_ emission concentrations from HVs. Although several studies have been performed using various ML techniques to predict several pollutants in conventional vehicles, to the best of our knowledge, this is the first study focusing on HV CO_2_ emission concentrations considering the entire set of conditions and challenges derived from the complete use of a HV (i.e., without excluding any driving scenario). By accurately predicting CO_2_ emission concentrations, this model could help in democratising the access to CO_2_ concentration information in HVs and in the development of strategies to reduce vehicle emission to combat air pollution. In addition, the proposed UWS-LSTM model has been embedded and deployed in a real-world HV to properly demonstrate its practical application in a low-powered Internet of Things (IoT) device, providing an efficient, cost-effective solution without the need for using PEMS. As seen from previous studies, IoT devices are increasingly improving their capabilities, which, coupled with the optimisation of ML techniques, can drastically provoke a change in this paradigm from highly-capable workstations to low-powered devices [13]. The outcomes of this study have the potential to assist policy makers and vehicle manufacturers regarding CO_2_ emission in HVs. As a result, our contributions are summarised as follows:

- Collection of a comprehensive dataset containing operational and emission-related information of a HV in real driving conditions and scenarios.
- Study of the most influential features presented in the dataset for prediction of CO_2_ emission concentrations.
- Design, implementation, training and validation of an accurate lightweight LSTM-based model (UWS-LSTM) able to perform CO_2_-concentration prediction for HVs.
- Comparison of traditional and advanced ML models in terms of accuracy, speed and model size with the proposed UWS-LSTM model.
- Deployment of the designed model in a low-powered IoT device installed within the vehicle to perform real-time on-road CO_2_-concentration prediction.

The rest of the paper is organised as follows. Section 2 elaborates the data collection and cleaning process, and addresses the input feature selection of the models evaluated. Moreover, it describes the definition of the proposed LSTM model and presents the execution environment. Subsequently, Section 3 explains the results found during the evaluation of each of the models evaluated. Section 4 concludes the paper.

## 2. Materials and Methods

**Figure 1:**
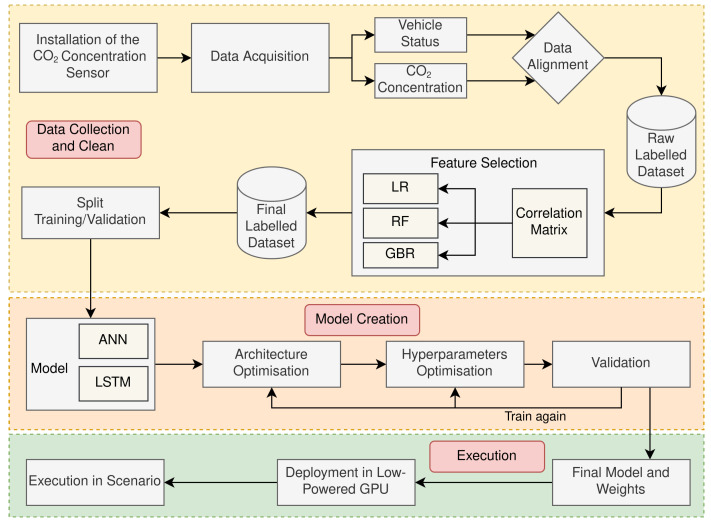
Workflow of the complete process from when the dataset was collected from the vehicle to the final execution, including designing and training of the ML model.

This sections explains the main stages addressed to achieve the contributions highlighted in the introduction. Figure 1 depicts the process followed to perform these stages. First, a data collection and cleaning process was carried out to gather the dataset that was used to train and evaluate the ML models. During this process, a study was conducted to investigate the correlation and effect of different input parameters sets on the assessed ML models. As a result, a subset of parameters present in the original dataset was identified as the final dataset and used in subsequent stages of the study. Later, a model creation stage was conducted with the objective of defining, training and validating the ANN-based ML models. Lastly, during the execution stage, an evaluation of each ML model was carried out with various performance indicators when these models were executed in a IoT device installed in a real vehicle. Further descriptions of the tasks involved in the defined stages are elaborated below.

### 2.1. Machine Learning Algorithms Evaluated

ML is a frequently used method to be applied for classification and regression in myriad of applications. In our study, 5 different ML algorithms were developed and tested to assess their performances when predicting the CO_2_ concentrations in HV exhausts:

- Linear regression: Linear regression tries to find the relationship between two variables by fitting a linear equation to observed data. One variable is the explanatory variable, and the other is the dependent variable [14].
- Random forest: Random forest regression is a supervised learning algorithm using ensemble learning method to perform regression. Ensemble learning method is a technique that each ML algorithm makes its own individual prediction. These predictions are then averaged to produce a more robust result than a single model [15].
- Gradient boosting trees: Gradient boosting decision trees are ensembles of several decision trees which are combined using loss functions to form a strong and effective model. The gradient boosting algorithm uses the gradient descent method to find the optimal point by minimising loss function.
- Artificial neural network (ANN): An ANN is based on a collection of connected nodes inspired by biological neural networks in the brain. The connection between any two neurons has some weight, which determines the effect of one neuron on the other. ANNs can be used to discover nonlinear and complex relationships among input and output variables [16].
- Long short-term memory: LSTM is a special form of a recurrent neural network (RNN) that solves the problem of vanishing gradient in vanilla RNNs [17]. LSTM models can store information to deal with time-series data due to their memory structure. In the LSTM model, a gating mechanism stores a long sequence of data and uses some information from previous steps to produce the output [18].

### 2.2. CO_2_ Concentration Collection

For the entire process of dataset collection, a SprintIR-R 20 (Gas Sensing Solutions Ltd., Cumbernauld, UK) sensor was used for measuring and recording the vehicle’s exhaust CO_2_ concentration, expressed in parts per million ppm. More specifically, the Sprint-IR sensor was found to be ideal for this use case, since it is able to report CO_2_ concentration values of up to 20% with an accuracy of ±70 ppm and a resolution of 10 ppm at a maximum sampling frequency of 50 Hz via non-dispersive infra red (NDIR) technology.

During the dataset collection process, the sensor was installed in the rear end of a vehicle and connected to the vehicle’s exhaust pipe. Furthermore, a set of tools were used to separate impurities and water vapour from the CO_2_ analyte, which included a hydrophobic filter, a water trap and a particulate filter to avoid any incorrect or inaccurate measurements. The CO_2_ collection system also featured an electric pump that helped the system to have a constant 0.5 l/min sample flow rate through the sensor’s measurement chamber. Moreover, the CO_2_ sensor was directly connected to an ESP32 micro-controller (Espressif Systems, Shanghai, China) via serial connection that performed measurements requests at a rate of 5 Hz. Before each drive, the sensor was calibrated manually using the external air as a reference, assuming a CO_2_ concentration value of 400 ppm. More information about the CO_2_ sensor can be found in the Supplementary information section of this manuscript.

### 2.3. Vehicle Status Data Collection

**Figure 2:**
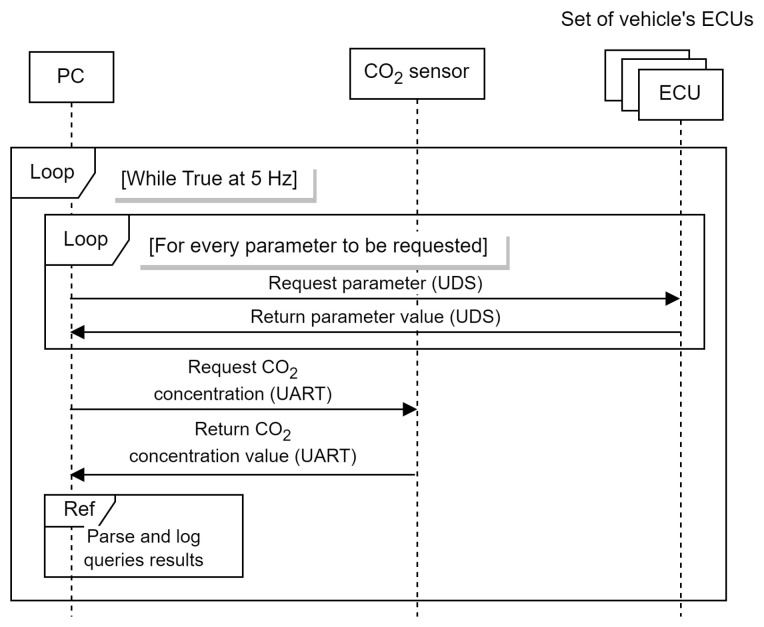
Data collection sequence diagram. Notice that after the acquisition of every ECU parameter and the CO_2_ concentration, the received messages have to be re-scaled and transformed from hexadecimal to decimal notation.

The collection of the onboard vehicle status parameters was carried out with custom software executed on a laptop that was connected to the vehicle’s CAN bus via its OBD2 port. The software was written in Python and performs UDS-like (Unified Diagnostic Services) [19] queries to periodically report a list of given parameters obtained from the vehicle’s electronic control units (ECU) at a fixed rate. The collection of parameters was done at 5 Hz; we stored the information recorded on separate files and drives and then merged it together. Figure 2 illustrates the sequence diagram of the data collection process.

The set of parameters acquired from the vehicle’s CAN bus comprises the following 21 parameters: accelerator position (%), vehicle speed (MPH), external atmospheric pressure (psi), engine load (%), coolant temperature (°C), acceleration (m/s^2^), distance travelled since vehicle startup (miles), drive mode, engine exhaust flow rate (kg/h), engine mode, engine speed (RPM), EV mode status, engine power delivered (kW), hybrid battery SOC (%), mass air flow (g/min), electric motor power delivered (kW), electric motor revolution (RPM), electric motor torque (Nm), GPS latitude, GPS longitude and altitude (m). Among the listed variables, there are 3 categorical parameters:

- Drive mode: indicates whether the power train source is hybrid or electric.
- EV mode status: in case the drive mode is electric, it indicates whether the electric mode is normal or “city”, which limits the power capacity up to 53 kW.
- Engine mode: indicates the status of the engine, such as (1) stopped, (2) stopping, (3) starting or (4) started.

### 2.4. Selected Input Parameters

Specific input features selection was conducted to identify a subset of input features that are most pertinent to the output variable and also to eliminate unnecessary information in the prediction process. To perform the input selection, the significance of the input data and the correlation amongst features should be estimated. Hence, to identify the most relevant parameters for this use-case, the correlation coefficient technique and some traditional ML algorithms were used for different parameters of the dataset.

#### 2.4.1. Correlation Matrix

**Figure 3:**
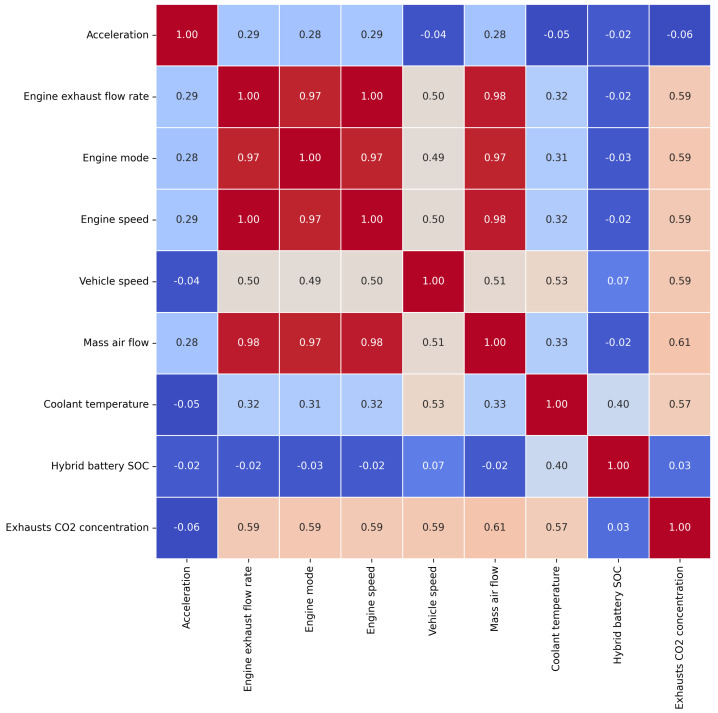
Correlation matrix composed of the final input values selected.

The first attempt to identify the most useful parameters was to calculate their correlation coefficients. Firstly, a correlation matrix was created with the entire set of parameters described in Section 2.3. The technique implemented was the Spearman-based correlation matrix, which provides a correlation coefficient based on the statistical dependence of ranking between two variables and covers non-linear relationships. The parameters with a high correlation score among them were eliminated, and the ones with a high correlation with the CO_2_ concentration were kept. Based on the observations seen in the correlation matrix, the parameters accelerator position, external atmospheric pressure, engine load, distance travelled since vehicle startup, drive mode, EV mode status, engine power delivered, electric motor power delivered, electric motor revolution, electric motor torque and altitude were removed, since they were considered less influential for the prediction. In addition, GPS coordinates of the car, i.e., latitude and longitude, were also discarded from the dataset, since the location of the car should not be a decisive factor in the prediction. As a result, Figure 3 presents the correlation matrix featuring the eight final selected input parameters and the output parameter to be predicted. Moreover, the resulting input parameters are presented and listed in Table 2.

#### 2.4.2. ML-Based Feature Selection

To further evaluate the impacts of the remaining input parameters, 255 different combinations of input values (the combination for 8 input values) were used to train and test each ML model to assess the adjusted R^2^ accuracy level achieved. Three distinct traditional ML algorithms were selected: linear regression (LR), random forest (RF) and gradient boosting regression (GBR).

Table 3 illustrates the top 4 combinations of input parameters that showed the best result for each ML algorithm tested. As seen, for both RF and GBR, the set of parameters that achieved the highest scores were: acceleration, hybrid battery SOC, vehicle speed, engine mode, coolant temperature and engine speed, leaving behind mass air flow and engine exhaust flow rate. However, LR showed that using all the values yielded the best score. In this case, as the RF and GBR obtained substantially higher accuracy than LR, the combinations of input parameters indicated by the best GBR and RF scores were chosen as candidates for the set of most influential parameters. As a result, two different set of input parameters were defined and used in the following stages of this research:

- Input set 1. Features 8 input parameters: acceleration, hybrid battery SOC, vehicle speed, engine mode, coolant temperature, engine speed, mass air flow and engine exhaust flow rate.
- Input set 2. Features 6 input parameters: acceleration, hybrid battery SOC, vehicle speed, engine mode, coolant temperature and engine speed.

### 2.5. Final Dataset

A total of 70,683 samples were collected from 235 min of driving. Three heterogeneous road scenarios are included in this dataset: motorway roads, urban roads and intercity roads; all of them were recorded at different times of the day. The classification of the samples in each road was done manually based on the GPS coordinates of the vehicle at each moment. As is common, 80% of the samples were employed for the purpose of training (56,546 samples in total), and the remaining 20% (14,137 samples in total) were used for validation and testing (10% and 10% respectively).

**Figure 4:**
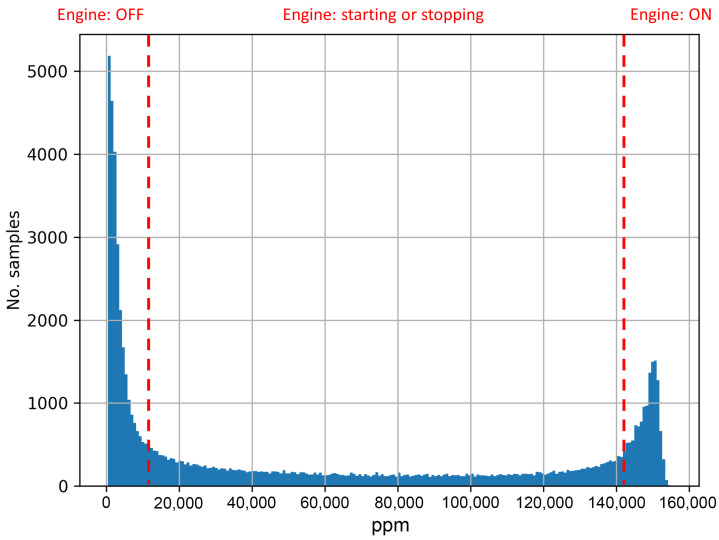
Histogram of CO_2_ concentration (ppm) present in the dataset.

The distribution of CO_2_ concentration in ppm in the dataset is represented in Figure 4. As is apparent, most of the samples in the dataset lie between the minimum and the maximum concentration values ppm. To the best of our knowledge, no other studies in the literature used the CO_2_ concentration emitted by HVs in combination with the vehicle’s operational performance indicators. In other datasets mentioned in the literature, the ppm concentration remains either constant when using a petrol engine or fluctuates within a limited range (usually between 80,000 and 120,000 ppm) when using a diesel engine [20,21]. With a hybrid engine, this is distributed along the entire range of possible values from 400 to approximately 155,000 ppm, generating the U-shaped distribution presented in Figure 4. This graph shows two peaks being captured over the data collection. First, a large portion of low-ppm measurements are captured when the engine is turned off (53.73%). Secondly, a high portion of high-ppm measurements are captured when the engine is on (38.41%). The remaining values correspond to switch-overs from the electric mode to the hybrid mode and vice versa. Therefore, these data are minimal, since it only takes a few seconds to make the transition (1.52% and 6.32% of the total dataset when the engine is starting and stopping, respectively).

The high frequency of switch-overs recorded during the drives carried out, which is very usual and normal behaviour in HV, and a non-constant CO_2_ concentration data distribution between both ends of the data, indicate the complexity and uniqueness of the dataset. This rapid change in concentration values during the switch-overs can be challenging for traditional ML algorithms. For this reason, more complex prediction mechanisms techniques were developed and used in this research, this being one of the main contributions of this manuscript.

#### Dataset Preprocessing

For training and validation, the complete dataset was standardised to prevent inputs with higher values from dominating those with the smaller values. To this end, formulae described by Equation (1) were applied to the dataset, where $Z_{i}$ describes the standardised value of a sample *i*, $x_{i}$ describes the original value of a sample *i*, μ represents the mean value of the this parameter in the dataset, and σ is the standard deviation of this parameter in the dataset.

(1) $$Z_{i}=\frac{x_{i}-\mu }{\sigma }$$

### 2.6. Proposed LSTM

**Figure 5:**
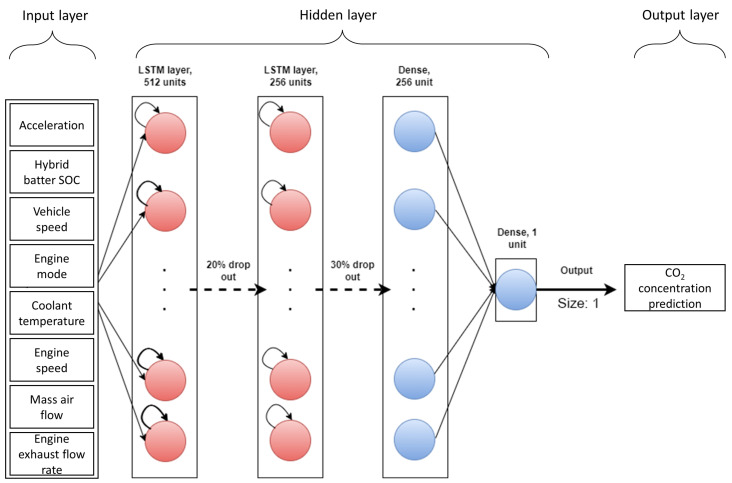
Architecture of proposed LSTM-based algorithm.

The proposed UWS-LSTM comprises three primary components: the first component is the input layer, which includes the most effective input parameters mentioned in Section 2.4.2. The second component is the hidden layers comprising two LSTM layers with 512 and 256 neurons in each LSTM cell. In this regard, a hierarchical structure of stacked LSTM layers has been shown to outperform single-layer LSTM models [22]. A drop out layer is also used after each LSTM layer with the probabilities of *p* = 0.2 and *p* = 0.3, respectively, to control the overfitting [23]. The second component also comprises two dense layers with 256 and 1 neurons, respectively. The third components is the output layer which outputs the CO_2_-concentration prediction as a single value. Figure 5 illustrates the architecture of the proposed algorithm, which outperforms the other algorithms mentioned in this article.

One of the advantages of using an LSTM model in comparison with the ANN model is that the former can be parameterized in terms of the amount of information that is taken into consideration from previous states. This term is often referred to as the lookback window. This parameter determines how many previous timesteps are considered to perform the current prediction. In order to figure out the influence of this parameter in the accuracy of the LSTM model, a process of model training and validation was conducted with a set of different lookback windows to compare the obtained scores. Further results can be observed in Section 3.1.1.

### 2.7. Hyperparameters

This subsection gathers the information regarding the configurations for the ML models studied in this work. Table 4 reflects the hyperparameter configurations. It is worth mentioning that for optimising the hyperparameters defined in the ANN model, a series of Bayesian optimisation iterations were conducted using keras-tuner.

### 2.8. Execution Environment

The complete prediction system was deployed in a real operating vehicle and tested on public roads to validate its performance under realistic conditions. The vehicle used was a Toyota Prius Plug-in Hybrid (2019). This vehicle features a 4-stroke, 4-cylinder 1798 cc gasoline engine that can output 90 kW of power. The maximum torque achieved is 142 Nm at 3600 RPM. As per the emissions, the vehicle is categorised under the Euro-class scheme as a Euro 6 DG type; according to official manufacturers information, the vehicle emits 28 g/km of CO_2_ (WLTP test).

The computing platform chosen was a Nvidia Jetson Xavier. This board is an off-the-shelf, low-consumption Linux-based system with a 512-core Volta GPU with Tensor Cores which runs at more than 21 tera operations per second (TOPS) and has 32 GB of memory. Different power consumption modes are supported: 10 W, 15 W and 30 W. Its portability and its power capability made it suitable for use cases to deploy our CO_2_-concentration predictor.

All the ANN-based algorithms were implemented and executed on TensorFlow 2. TensorFlow [24], which is a state-of-the-art ML platform that is fully compatible with Nvidia compute unified device architecture (CUDA); therefore, it was the selected platform for implementation and execution of the algorithms in Jetson Xavier. On the other hand, the python package Scikit-learn was also used to perform the predictions with the traditional ML algorithms due to it is simplicity and efficiency.

## 3. Results and Discussion

In this section, we present and analyse the results obtained from the evaluations of the ML models outlined in previous sections of the manuscript. The results are presented in two forms: quantitative and qualitative. The quantitative results consist of a set of numerical measurements, and the qualitative results pertain to the implementation and performance of the CO_2_ predictor within an actual vehicle environment.

### 3.1. Quantitative Results

The quantitative accuracy of the predictions was measured by calculating the errors given by adjusted R^2^, mean absolute error (MAE), mean square error (MSE) and root mean squared error (RMSE), described by Equations (2)–(5), respectively. In these equations, $R^{2}$ refers to the ordinary R-squared formula, *n* is the number of data points, *p* is the number of predictor variables in the model, $y_{i}$ is the true value of the *i*th observation and ${\hat{y}}_{i}$ is the predicted value of the *i*th observation.

(2) $$\mathrm{Adjusted}\;R^{2}=1-\frac{\left(1-R^{2}\right)\left(n-1\right)}{n-p-1}$$

(3) $$\mathrm{MAE}=\frac{1}{n}\sum_{i=1}^{n}\left|y_{i}-{\hat{y}}_{i}\right|$$

(4) $$\mathrm{MSE}=\frac{1}{n}\sum_{i=1}^{n}{\left(y_{i}-{\hat{y}}_{i}\right)}^{2}$$

(5) $$\mathrm{RMSE}=\sqrt{\frac{1}{n}\sum_{i=1}^{n}{\left(y_{i}-{\hat{y}}_{i}\right)}^{2}}$$

#### 3.1.1. LSTM Lookback Window

As previously mentioned in Section 2.6, a key hyperparameter to be defined for LSTM-based models is the lookback window. Table 5 presents the different results in terms of accuracy after training and validating the UWS-LSTM model with different lookback window sizes. In addition, we presented in Section 2.4.2 that two particular input parameters yielded different scores depending on the ML model used. For this reason, these input parameters were part of the analysis to determine the best LSTM configuration.

As observed in the results, configurations with smaller lookback window sizes, such as 1, 2 or 4, performed inferiorly. However, there was a significant increase in accuracy as the window size increased: an improvement of approximately 25% when using both input parameter sets, when comparing a window size of 8 to 16. Notably, the highest accuracy was achieved with a window size of 32 and input parameter set 1: an adjusted R^2^ score of 0.975, surpassing the same window size with input parameter set 2, which had a score of 0.9702. This illustrates that a sufficiently large window size can substantially enhance the accuracy of the model. Moreover, the selected input parameters for the training have effect on the results. Thus, input parameter of set 1 scored better results for every lookback window size configuration.

Nonetheless, it is also known that higher lookback windows demand higher computational resources. Since the aim of this research was the deployment of the developed model in a low-powered device, no bigger window sizes were considered for this study.

#### 3.1.2. Accuracy

**Figure 6:**
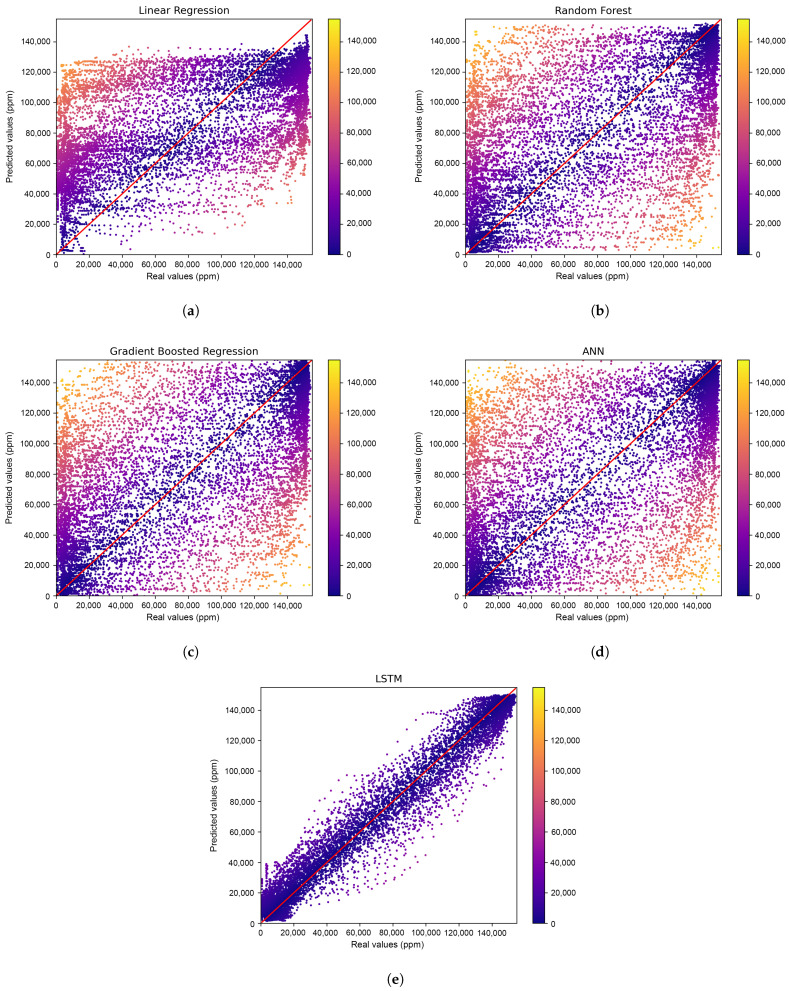
Prediction results. The colour-code is based on the difference between the original data and the predicted data. (**a**) Very dispersed predicted values with an R${}^{2}$ of 42%. (**b**) Better prediction in ppm edges. Still high dispersion. (**c**) GBR still failing in predicting the CO_2_ in switch-overs. (**d**) The ANN performed very poorly on power-train switch-overs, with an accuracy of 46%. (**e**) LSTM narrows all predictions to the ground truth.

Table 6 shows the accuracy obtained for each ML model. It is very important to clarify that, due to the fact that LSTM-based models are naturally designed to be trained (and executed) following a time-based order, for the sake of a fair comparison among all the models, the other 4 ML algorithms were trained following the same procedure, i.e., without shuffling the testing dataset. Figure 6 shows the prediction results of the models. The figures show the real values against the predicted values in ppm. In addition, the results are colour-coded based on the differences between these two values.

As evident in Table 6, the performance of the LR model was the lowest among all the ML models evaluated, with scores of 0.4214, 0.6566, 0.6021 and 0.7759 for adjusted R^2^, MAE, MSE and RMSE, respectively. This is also reflected in Figure 6a; a high degree of dispersion can be observed throughout the plot. In contrast, RF, GBR and ANN models exhibited similar accuracy levels. Furthermore, the distribution of the results, as seen in Figure 6b–d, illustrates a more accurate prediction on the edges of the dataset’s distribution, which correspond to moments when the engine was either started or stopped. However, a more dispersed result can be observed in all three models when predicting values associated with power-train switch-overs. This serves as clear evidence of how the presence of power-train switch-overs hinders the ability of these three models to predict the CO_2_ concentration in hybrid vehicles (HVs). Specifically, for RF, the scores obtained were 0.4645, 0.5507, 0.5572 and 0.7465 for adjusted R^2^, MAE, MSE and RMSE, respectively. For GBR, the scores were 0.4597, 0.5555, 0.5622 and 0.7498 for adjusted R^2^, MAE, MSE and RMSE, respectively. For ANN, the scores obtained were 0.4639, 0.5346, 0.5577 and 0.7468 for adjusted R^2^, MAE, MSE and RMSE, respectively.

Ultimately, the proposed UWS-LSTM algorithm demonstrated superior performance in comparison to the other evaluated methods, as evidenced by its adjusted R^2^ value of 0.975 and its MAE, MSE, and RMSE values of 0.1135, 0.0261, and 0.1616, respectively. Additionally, as demonstrated in Figure 6e, the LSTM algorithm shows exceptional accuracy not only at the edges of the data distribution, but also in the middle values corresponding to engine switch-overs, as depicted in Figure 4. This highlights the potential of LSTM-based models in predicting more complex non-linear relationships by utilising previous states.

#### 3.1.3. Latency

This subsection focuses on the experiments carried out after deploying and applying our predictive model in TensorFlow in the Nvidia Jetson board described in Section 2.8. In addition to the proposed UWS-LSTM model, LR, RF, GBR and ANN were studied to compare the different latency levels and assess the trade-offs between speed and accuracy. All these results were obtained by setting the Nvidia Jetson Xavier on different power modes: 10 W, 15 W and 30 W.

**Figure 7:**
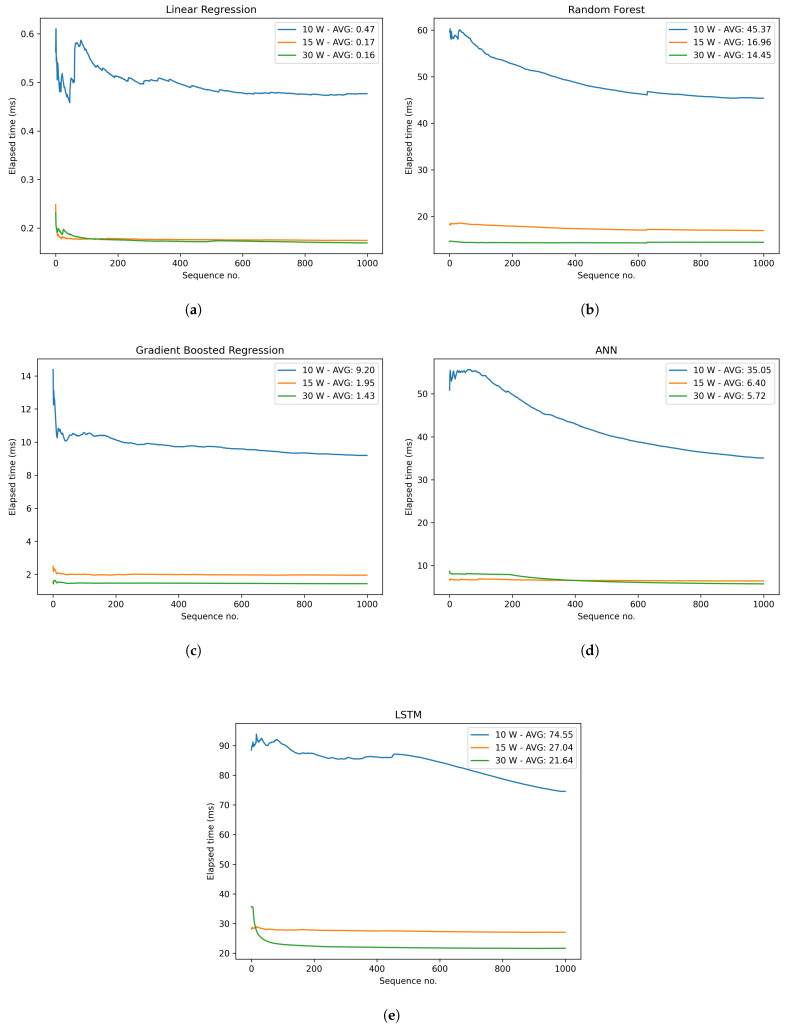
Performance evaluation. (**a**) Outstanding results in speed. (**b**) Good results at 15–30 W. (**c**) Less than 10 ms for each scenario. (**d**) Good for 15–30 W. (**e**) Achieved real-time for 15–30 W.

The results in Figure 7 are presented in milliseconds, which shows the cumulative average inference time for the first 1000 iterations. The cumulative average inference time was calculated following Equation (6), where *x* is the inference time at a time step *i* and *t* is the current time step. The inference time was calculated, since the input was fed to the algorithm until the results were provided in the output. Thus, 15 experiments were carried out to evaluate five algorithms in three different power modes. As LR, RF and GBR models were implemented with the Scikit learn package and not with TensorFlow, their execution was carried out by the CPUs available on the device. On the other hand, the TensorFlow framework was used to implement neural network-based algorithms, so they were compatible with the Jetson’s GPU.

(6) $$T_{t}=\frac{\sum_{i=1}^{t}x_{i}}{t}$$

First the performance levels of traditional ML algorithms, including LR, GBR and RF, were analysed and compared. The results, as depicted in Figure 7a–c, indicate that the LR algorithm performed with the lowest average cumulative latency of 0.16 ms per iteration. GBR exhibited a slightly slower performance, with an average execution time of 1.43 ms per iteration under a 30 W power consumption. On the other hand, RF algorithm demonstrated the highest average latency of 14.45 ms among the three traditional ML algorithms. Furthermore, ANN and LSTM models, as presented in Figure 7d,e, respectively, both exhibited slower average performance when operating at 30 W. Specifically, the ANN-based model recorded an average execution time of 5.72 ms, whereas the LSTM-based model took 21.64 ms on average. These results suggest that traditional ML algorithms generally exhibit faster performance compared to ANN and LSTM models. However, as seen in previous sections, there is a trade-off between speed and accuracy, which is especially notable with the UWS-LSTM model.

Additionally, as observed in every graph, there was a significant improvement in terms of speed when the Nvidia Jetson board was configured to operate at 15 W in comparison to 10 W. Oppositely, changing from 15 to 30 W did not reveal a remarkable enhancement in terms of latency.

#### 3.1.4. Model Size

**Figure 8:**
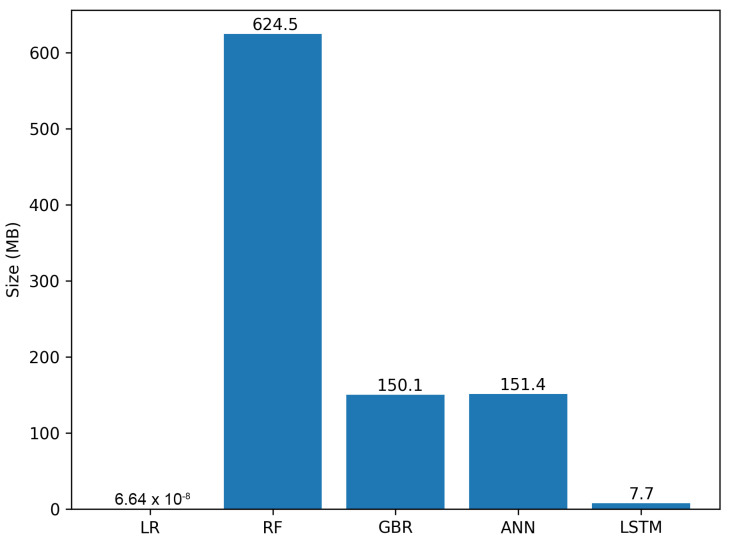
Comparison of the ML models size.

In an IoT setting, the size of a machine learning model is a crucial consideration. Due to the limited storage and computational resources of IoT devices, models with smaller sizes are typically more favourable, as they can be more easily exported and deployed on these devices. In this context, the UWS-LSTM model, with a size of 7.7 MB, can be considered relatively lightweight and well-suited for IoT applications. As depicted in Figure 8, among the five models presented, only the LR model had a smaller size than the UWS-LSTM model. However, as previously established through the analysis of other performance metrics, the LR model dud not present an appropriate trade-off between model size and accuracy. Thus, the UWS-LSTM model emerged as the most promising candidate for deployment on IoT devices.

### 3.2. Qualitative Results

**Figure 9:**
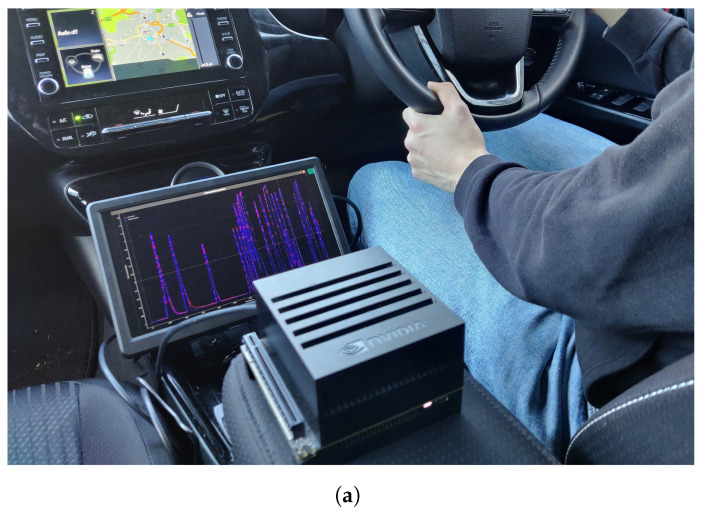

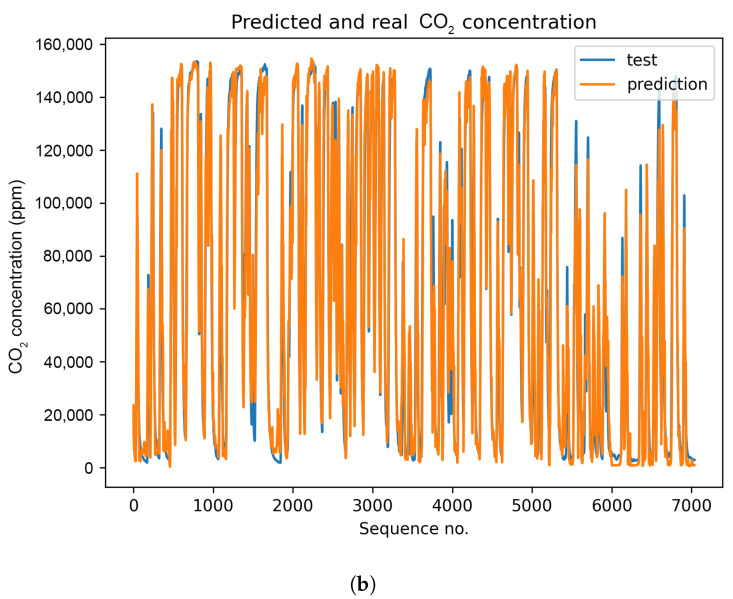
Vehicle onboard deployment. (**a**) Setup. (**b**) Prediction against real recorded values.

The UWS-LSTM model was instantiated and installed within a real scenario to prove the potential of its use under realistic scenarios. The Nvidia Jetson was set up in the Toyota Prius car described in Section 2.8. The installation can be seen in Figure 9a, where the display shows the real-time execution and CO_2_ measurement during the evaluation. Figure 9b presents the predicted concentration against the real CO_2_ values captured by the sensors during the installation and evaluation process. As can be seen, the prediction results fit to a great extent the real values, including when the switch-overs were performed.

During the on-board evaluation, the real-time predictions were shown on a display. However, IoT devices represent an ideal platform for remote monitoring, thanks to their easy integration with communication protocols over wireless networks, leveraging an incipient solution for remote ML-based emissions monitoring.

### 3.3. Discussion

In this paper, we have presented the design, implementation, training and validation of a LSTM-based ML model for prediction of CO_2_ concentration for HVs. Initially, a data gathering process was conducted with the aim of collecting features of a real-world HV and its CO_2_ emission concentrations. This dataset is the only of its kind, to the best of our knowledge, since it contains over 70,000 of more than 20 operational parameters and CO_2_ emission concentrations of a HV during 235 min of driving in different scenarios, regardless of the power-train source (i.e., data were collected when the engine was started and stopped).

To determine the relevance of the gathered data, we performed an input feature selection by calculating the correlation score among the dataset parameters and evaluating 765 different combinations of training with different input features in three traditional ML algorithms. This resulted in two sets of parameters, one with six parameters and another with eight parameters, which was better for each different algorithm, showcasing that no specific input parameter set can be foreseen as optimal for every ML model. This input feature selection procedure allowed identifying the most relevant parameters for predicting emissions and eliminating irrelevant or redundant data, which proved to improve the performance in the assessed models.

Moreover, we have designed and evaluated the proposed ML model, UWS-LSTM, for predicting CO_2_ emission concentrations in a HV. The results showed that this model outperformed other ML mechanisms (achieving 97.5% accuracy level), especially with high complexity of the labelling distribution observed during power-train switch-overs carried out by the HV (see Figure 4). This makes the UWS-LSTM model a valuable tool for researchers studying emissions in HVs, where the use of the engine is not constant, and therefore, the CO_2_ concentrations fluctuate greatly over time. In terms of the computational cost derived from the training and validation processes, the proposed UWS-LSTM was found as a relatively inexpensive and quick solution with 7.7 MB in size and 21.64 ms of inference time. The proposed model took an average of 30 min for training when utilising a dedicated GPU. It is hard to make a comparison with other approaches seen in the literature about the computational costs implied in the process, since this information is usually not disclosed by the authors.

Finally, we have implemented the UWS-LSTM model in a low-powered IoT device installed in the same vehicle where we gathered the data (see Figure 9). An evaluation of its performance with three different power settings was conducted as recorded to measure the speed. As observed, the UWS-LSTM reached a minimum average execution time of 21.64 ms, which was considerably more than the second best ML algorithm (GBR with 1.43 ms). Nonetheless, as for the model sizes, the proposed UWS-LSTM was the second most lightweight model, being 7.7 MB; the other three most accurate models were 150.1, 151.4 and 624.5 MB, respectively. Overall, the proposed UWS-LSTM seems to better fit the performance and weight requirements for being implemented in low-resource environments.

In conclusion, the work presented in this study would make an important contribution to the field of HV emission concentration prediction. The large collected dataset, the effective input feature selection and the performance of the UWS-LSTM model make it a valuable resource for researchers in the area of ML-based pollution prediction, which can greatly help the development of new cost-effective emission-aware driving strategies. Moreover, thanks to the use of IoT devices, this solution can potentially facilitate the transmission of CO_2_ emission concentrations over wireless networks.

Despite the provided results proving the capacity of the model to accurately predict the concentrations with a decent extent of success, we are aware of the shortcomings in execution time. Therefore, further research will be conducted towards reducing the execution time for the proposed model.

## 4. Conclusions

This paper presented the design and implementation of an LSTM-based ML model for predicting a CO_2_ emission concentration in HVs. A dataset of over 70,000 data points of more than 20 operational parameters and CO_2_ emission concentrations of a HV over 235 min of driving in different scenarios was gathered, and an extensive input feature selection was conducted to improve the performances of the ML models. The proposed UWS-LSTM model outperformed the other ML mechanisms, achieving 97.5% accuracy, including in scenarios where the engine usage is not constant. The proposed model was implemented in a low-powered IoT device in a real vehicle and proved to have a good balance of performance and latency. It is believed that this work makes a valuable contribution to the field of HV emission concentration prediction and can aid in the development of new cost-effective emission-aware driving strategies and exhaust monitoring.

## Figures and Tables

**Table 1:** Comparison of prediction techniques for vehicle CO_2_ emissions.

Ref.	Prediction Target	Vehicle/ Engine Type	Fuel Type	Algorithm	Framework	Exec. Env.	Accuracy (%)	Exe. Speed (ms)	Model Size (MB)
[3]	CO_2_ conc. (ppm)	ng	ng	GBR	ng	ng	91	ng	ng
[4]	CO_2_ emission rate (g/s)	Passenger/ ICE	Diesel	GBR	Scikit-learn	ng	99	ng	ng
[5]	CO_2_ emission rate (g/s)	Passenger/ Hybrid	Gasoline	XGBoost	Scikit-learn	ng	89.8	ng	ng
[6]	CO_2_ emission rate (g/km)	Passenger/ ICE	Gasoline	LSTM	ng	PC	9.30 (RMSE)	ng	ng
[7]	CO_2_ emission rate (g/s)	Passenger/ Hybrid	Gasoline	GPR	ng	ng	69	ng	ng
[8]	CO_2_ emissions (kg)	Passenger/ ICE	Gasoline	LR	ng	ng	95.75	ng	ng
[9]	CO_2_ conc. (%)	ng/ICE	Diesel	PA-LSTM	ng	PC	94.6	ng	ng
[10]	CO_2_ conc. (ppm)	Construction/ ICE	Diesel	RF	ng	ng	94	ng	ng
[11]	CO_2_ emissions (kg)	Passenger/ ICE	Diesel	ANN	TensorFlow	ng	0.5 (RE)	ng	ng
TP	CO_2_ conc. (ppm)	Passenger/ Hybrid	Gasoline	LSTM	TensorFlow	Nvidia Jetson Xavier	97.5	21.64	7.7

TP = this paper; ICE = internal combustion engine; ng = not given; RE = relative error.

**Table 2:** Statistical values for each of the input features selected in the final dataset.

ID	Name	Unit	Mean	Min.	Max.
1	Acceleration	m/s^2^	0.011	−7.058	4.634
2	Hybrid battery SOC	%	16.63	10.58	78.55
3	Vehicle speed	MPH	32.61	0	79.535
4	Engine mode	n/a	n/a	n/a	n/a
5	Coolant temperature	°C	83.24	23	93
6	Engine speed	RPM	730.734	0	4512
7	Mass air flow	g/min	424.836	25.2	3753.6
8	Engine exhaust flow rate	kg/h	26.074	0	242.2

**Table 3:** Top 4 most accurate combinations of different input parameters with three traditional ML algorithms. The black dots indicate that the parameter was included in the input parameter for that combination, whereas the white dots indicate the opposite.

ML Algorithm	Input Parameters								Adjusted R^2^ Score
	Acceleration	Hybrid Battery SOC	Vehicle Speed	Engine Mode	Coolant Temperature	Engine Speed	Mass Air Flow	Engine Exhaust Flow Rate	
LR	●	●	●	●	●	●	●	○	0.518
	●	●	●	●	●	●	●	●	0.518
	●	●	●	●	○	●	●	○	0.517
	●	●	●	●	○	●	●	●	0.517
RF	●	●	●	●	●	●	○	○	0.91
	●	●	●	○	●	●	●	○	0.91
	●	●	●	●	●	●	●	○	0.909
	●	●	●	●	●	○	●	○	0.908
GBR	●	●	●	●	●	●	○	○	0.916
	●	●	●	○	●	●	○	○	0.913
	●	●	●	●	●	○	○	●	0.909
	●	●	●	●	●	○	●	○	0.909

**Table 4:** Final hyperparameter configuration for each ML model.

Algorithm	Input Set	No. Estimators	Loss Function	Max. Depth	Min. Samples Split	Max. Leaf Nodes	Subsample	Learning Rate	Epochs	Batch Size	Optimiser	Lookback Window Size
LR	1	n/a	n/a	n/a	n/a	n/a	n/a	n/a	n/a	n/a	n/a	n/a
RF	2	800	MSE	None	2	None	n/a	n/a	n/a	n/a	n/a	n/a
GBR	2	800	MSE	12	10	n/a	0.8	0.08	n/a	n/a	n/a	n/a
ANN	1	n/a	MSE	n/a	n/a	n/a	n/a	$1\times 10^{-3}$	1000	32	Adam	n/a
UWS-LSTM	1	n/a	MSE	n/a	n/a	n/a	n/a	$1.6\times 10^{-4}$	100	32	Adam	32

n/a: not applicable.

**Table 5:** Accuracy score for each LSTM network configuration.

(a) Input Parameters Set 1		(b) Input Parameters Set 2	
**Lookback Window**	**Adjusted R^2^**	**Lookback Window**	**Adjusted R^2^**
1	0.5627	1	0.5585
2	0.5015	2	0.4895
4	0.4738	4	0.4446
8	0.6036	8	0.5237
16	0.8389	16	0.7901
32	0.975	32	0.9702

**Table 6:** Accuracy scores obtained with the complete test dataset. Note that the scores shown refer to standardised data (see Equation (1)).

Algorithm	Adjusted R${}^{2}$	MAE	MSE	RMSE
LR	0.4214	0.6566	0.6021	0.7759
RF	0.4645	0.5507	0.5572	0.7465
GBR	0.4597	0.5555	0.5622	0.7498
ANN	0.4639	0.5346	0.5577	0.7468
UWS-LSTM	0.975	0.1135	0.0261	0.1616

## Data Availability

Data available on request due to privacy restrictions.

## Supplementary Materials

Video of instantaneous CO_2_ pollution monitoring: https://beyond5ghub.uws.ac.uk/vehicle_emissions_monitoring/ (accessed on 17 November 2022).

## Abbreviations

The following abbreviations are used in this manuscript:

ANN	Artificial Neural Network
CAN	Controlled Area Network
cc	cubic centimeters
CNN	Convolutional Neural Network
CO_2_	Carbon Dioxide
CPU	Central Processing Unit
CUDA	Compute Unified Device Architecture
ECU	Electronic Control Unit
GBR	Gradient Boosting Regression
GPS	Global Positioning System
GPU	Graphics Processing Unit
HV(s)	Hybrid Vehicle(s)
ICE	Internal Combustion Engine
IoT	Internet of Things
LR	Linear Regression
LSTM	Long Short-Term Memory
MAE	Mean Absolute Error
ML	Machine Learning
MLP	Multi Layer Perceptron
MPH	Miles per Hour
MSE	Mean Square Error
NDIR	Non Dispersive Infra Red
NO_x_	Nitrogen Oxide
PEMS	Portable Emissions Monitoring System
ppm	parts per million
psi	pounds per square inch
RF	Random Forest
RMSE	Root Mean Square Error
RNN	Recurrent Neural Network
RPM	Revolutions per Minute
SOC	State of Charge
SVR	Support Vector Regression
TOPS	Tera Operations per Second
UDS	Unified Diagnostic Services
W	Watts
WLTP	Worldwide Harmonised Light Vehicle Test Procedure
XGBR	Xtreme Gradient Boosting Regression
